# Not to Be Given during Pregnancy

**Published:** 1895-10

**Authors:** 


Not to be Given During Pregnancy.—The chief remedies which are dangerous to the pregnant woman are salicylate of soda and ergot. Purgatives—castor oil, mineral salts, and especially aloes should be avoided. Quinine in too large doses should also be omitted; when given at all it should be guarded by combination with opium.—*Hugenin.*

